# Incidence and Risk of Lung Cancer in Tuberculosis Patients, and Vice Versa: A Literature Review of the Last Decade

**DOI:** 10.1155/2022/1702819

**Published:** 2022-12-19

**Authors:** Sutapa Bhowmik, Nayan Chandra Mohanto, Dipanjon Sarker, Asma Ahsan Sorove

**Affiliations:** ^1^Department of Microbiology, Noakhali Science and Technology University, Noakhali 3814, Bangladesh; ^2^Department of Biochemistry and Molecular Biology, School of life Sciences, Shahjalal University of Science and Technology, Sylhet 3114, Bangladesh

## Abstract

**Background:**

The incidence and risk of both lung cancer (LC) and tuberculosis (TB) are increasing rapidly. These two diseases frequently exist together and can influence the incidence and risk of each other. The aim of the current review was to summarize the incidence and risk of LC in TB patients, and vice versa, short out research gap, and contemplate future research perspectives. *Methodology*. PubMed and Scopus databases, and Google Scholar search engine were searched for epidemiological studies that investigated the incidence and risk of TB and LC, published since January 2011 to April 2022, and written in English. We used the searching keyword “tuberculosis” combined with “lung cancer” and associated medical subject heading (MeSH) to retrieve eligible research articles. We retrieved information's regarding the diagnosis of TB and LC, confounders, the associations of TB and LC, and incidence and risks of each other.

**Results:**

We found higher incidence rate and risks (1.64 to 6 times higher) of LC in TB patients in comparison to non-TB participants. However, the incidence rate and risks of TB in LC patients were comparatively low. Male patients were exhibited higher risks than female. The medical comorbidities, smoking habits, and age can also influence the associations and risks of LC in TB patients or vice versa.

**Conclusion:**

Our summarized studies might suggest that existing active TB may increase the incidence and risk of LC. However, large prospective cohort study is warranted to explore the real scenario worldwide.

## 1. Background

Lung cancer (LC) is the major global health concern and is associated with substantial morbidity and mortality [1–4]. Comparatively, more people die from LC than of colon, breast, and prostate cancers combined worldwide [3]. LC has shown a continuously increasing trend in many countries, and over the time, became the biggest cancer killer of men throughout the world from the rarest form of diseases [5]. Only in 2012, over 1.6 million of people died due to LC. There are two main types of LC including non-small-cell LC (adenocarcinoma, squamous cell carcinoma, and large cell carcinoma) and small-cell LC [6]. There are several biochemical, genetical and epigenetic, immunological, and epidemiological factors associated with the development of LC [7–9]. The coexistence of some medical conditions and diseases especially tuberculosis (TB) also reported to be associated in the pathogenesis of LC. The incidence and risk for the development of LC have been reported to be increased in patients with both latent TB and active TB [10–13].

Accordingly, TB has killed more humans than any other pathogen, after prolonged coevolution to optimize its pathogenic strategies [14]. An estimated 10 million people were infected with TB worldwide in 2020 irrespective to countries and age groups [15]. It is the top global infectious pandemic after coronavirus disease 2019 (COVID-19) caused by the pathogen *Mycobacterium tuberculosis* [14]. The latent TB infection (LTBI) describes the condition wherein an individual is infected with bacteria but not currently manifesting active disease [16, 17]. Nearly one-fourth of the world's population has an LTB infection [15]. Active TB develops in approximately 10% of people with an LTB infection [18, 19]. On the other estimate, people with active TB can infect 5–15 other people through close contact over the course of a year. When people with TB cough, sneeze, or spit, they propel the TB germs into the air and person who inhaled the TB germ-contaminated air is subsequently infected [15]. Active TB is a chronic inflammatory reaction of pulmonary parenchyma that causes irreversible fibrosis and scarring, accompanied by an impaired immune response [20–22]. Chronic TB infections cause the accumulation of genomic changes, and exposure to growth factors can cause multistep cellular transformation, leading to dysplasia and malignant squamous cell carcinoma [23]. Human susceptibility to TB is also associated with polymorphisms of several genes related to innate immunity and the inflammatory response, such as Toll-like receptors, tumor necrosis factors, and inducible nitric oxide; notably, these genes are associated with cancer susceptibility [23–26]. Depending on mechanistic pathways, time required for the development of LC in TB patients is varied, and frequently depends on sociodemographic and lifestyle factors along with some medical comorbidities.

On the contrary, the American Thoracic Society and the Center for Disease Control and Prevention have recognized that LC increases the risk of TB. Patients with LC may exhibit deficiencies in cell-mediated immunity as either a direct effect or an indirect effect related to chemotherapy and consequently developed TB [6, 27]. Some studies also reported the development of TB in LC patients [28–30]. So, a significant level of controversies still exists regarding the association of TB and LC which need to be explored. Accordingly, the coexistence of TB and LC has created great attention to epidemiologist, physician, and clinical researchers since both become deadly. Previously, a few reviews explored the prevalence of LC in TB patients, facts and fiction, and management of coexisting TB and LC [31–34]. However, almost all were systematic reviews and meta-analysis based on single outcome (either TB or LC) that did not explore the worldwide current incidence and risk scenario of TB in LC patients, and vice versa. Therefore, the objectives of the current review were to summarize the study that investigated the incidence and risk of LC in TB patient, and vice versa; to find out potential knowledge gap; and to outline future research perspectives.

## 2. Methodology

### 2.1. Database Searching

A systematic search was made in PubMed and Scopus databases, and Google scholar search engine for relevant publications that investigated the associations between TB and LC especially the incidence and risk using the keyword “tuberculosis” AND “lung cancer” and associated medical subject headings (MeSH) to retrieve original research articles of human epidemiological studies. Additional filtering was performed to customize the research based on article type (journal articles), study subjects (human), language (English), and publication year (January 2011 to April 2022). The references of the retrieved research articles were also searched for relevant publications.

### 2.2. Article Selection

The full-length original research work from all over the world regardless of gender, age groups, and race/ethnicity was included in this review. Inclusion criteria were as follows: (1) epidemiological study that investigated the associations between LC and TB, and evaluated the incidence and risk of LC in TB patients, and vice versa; (2) all ages and/or life stage of disease conditions; (3) outcome either TB or LC; and (4) article must be written in English and published in between January 2011 and April 2022. Information regarding study design, country, number of study subjects, follow-up period of disease development (both endpoints), diagnosis (both LC and TB), covariates or confounders, and incidence and risk of LC in TB patients, and vice versa were retrieved and summarized (Table 1). All the articles addressing case reports, clinical trials, and treatment strategies or intervention were excluded. Finally, a total of 24 articles that fulfill our inclusion criteria were selected and summarized in this review (Table 1). Last two authors of the present study searched and retrieved the existing relevant research articles, and first author cross-checked to minimize the selection bias. We did not select articles based on only positive association rather considered all possible associations (positive, negative, and null).

## 3. Results

### 3.1. Study Design, Participants, and Disease Diagnosis

We summarized a total of 24 human epidemiological studies from the last decades including cross-sectional, case-control cohort and retrospective cohort studies that explored the interrelationship between TB and LC incidence, risk, and mortality (Table 1). The study subjects enrolled in these studies were either adults or elderly with the age ≥ 20 years except one study that recruited participants ≥ 15 years old. The number of total participants was varied among studies, and ranged from 63 to 1607710. Among the 24 studies, LC was the outcome in 16 studies, whereas TB was the outcome in the remaining 8 studies. In the cohort studies, follow-up periods were varied from 1 to 19 years. Most of the studies diagnosed the TB by chest X-ray (CXR) or bacteriological confirmation via microscope, and LC by CXR, CT scan, and PET biopsy and identified based on ICD-9 or 10. All the studies adjusted potential confounders or covariates to strengthen their findings. Major considering covariates were age, sex, smoking, drinking alcohol, and medical comorbidities.

### 3.2. Incidence and Risk of LC in Patients with TB

All of the cohorts that investigated the risk of LC in TB patients recruited TB participants as case groups and general or non-TB (may exist other disease) individuals as control groups (Table 1). One recent study recruited chronic obstructive pulmonary disease (COPD) and non-COPD participants with and without history of pulmonary TB [35]. The incidence rate ratio (IRR) was varied among studies based on follow-up periods [36–42]. The highest IRR (11-fold higher in TB participants than non-TB participants) was found in a case-control cohort of Taiwanese participants (26.3 vs. 2.41 per 10000 participants) in a total 9-year period of follow-up [41]. On the other hand, the lowest IRR was 1.76 in a total 10-year of follow-up [40]. Accordingly, the IRR highly depends on follow-up duration and was relatively higher in short periods of follow-up than that in long periods [12, 28, 36, 38, 39].

Several studies investigated the relative risk of LC in TB patients. Ho et al. reported approximately 2 times higher risk of secondary LC in TB patients compared to general study participants in Taiwan, whereas other two studies reported 1.64 and 4.37 times higher risk of LC in TB patients [12, 40, 41]. Two separate studies conducted in South Korea in 2020 consequently reported approximately 4 and 6 times higher risk of LC in TB patients than that of control participants, respectively [13, 36]. Other three European studies from Denmark, Netherlands, and Finland were also found 2.24, 1.75, and 2 times higher risk of LC in TB patients, respectively [28, 39, 43]. A few studies found higher mortality and lower survival rate of LC in TB patients compared to non-TB patients [44–46]. Some studies showed sex-specific incidence and risk of LC and stated that men participants had higher risk than that of women participants [13, 36, 47] except one study that reported higher adjusted hazard ratio [6] in women than men [42]. Age-dependent incidence and risk of LC also vary among studies, and most of them showed higher risk in the oldest participants compared to relatively lowest aged participants [36, 37, 42].

Lack of medical facilities and/or treatment and medical comorbidities were also reported to be associated with the increased risk of LC in TB patients [12, 41]. Very recently, one study conducted in South Korea by recruiting both COPD and non-COPD patients with and without PTB showed that COPD patients with baseline history of PTB had higher incidence rate of LC in comparison with COPD patients without PTB [35]. The aHR for LC was significantly higher in COPD patients with PTB in comparison to without PTB, whereas the aHR for LC was not different between PTB and without PTB among non-COPD patients [35].

Most of the studies that investigated the effect of smoking in LC development in TB patients found increased incidence and mortality risk of LC in smoker compared to nonsmoker [13, 37, 47, 48]. However, a recent study reported that never smokers with COPD had higher risk of LC incidence in the participants with PTB compared to those without PTB. In ever smokers (past or current smoker) with COPD, the corresponding risk was null [35].

### 3.3. Incidence and Risk of TB in Patients with LC

All the existing study that investigated the risk of active TB/LTB in LC patients recruited LC participants as case groups and general or non-LC (may exist other disease) individuals as control groups (Table 1). In 2021, two studies conducted to investigate the incidence and risk of TB in LC patients. Abdelwahab et al. reported that 25% LC patients in Egypt developed LTB with no effect of age, sex, and smoking along with other medical comorbidities [49]. Another study conducted in Canada reported the increased risk of active TB (aHR = 11.20) after 19 years of follow-up [50]. One retrospective study which investigated the incidence and risk of clinically diagnosed TB and bacteriologically confirmed TB (BCTB) in LC patients found the BCTB IRR was 50.35 in comparison with control [51]. One retrospective study in Thailand followed up 18 years and found a cumulative incidence 1.15% for TB in LC patients [52]. Suzuki et al. investigated the cumulated incidence of TB in Japanese LC patients at different follow-up stages and found 0.65%, 1.15%, and 1.38% incidence of TB at 0.5-, 1- and 2-year time periods [53]. Two cohort studies conducted separately in Taiwan evaluated higher TB risk and mortality risk in patients with LC that developed TB in comparison to controls [44, 54]. Wu et al. found higher incidence and risk of TB in LC patients and estimated that the average interval from diagnosis of LC to active TB development time was 748 days [55].

## 4. Discussion

In this literature review, we summarized the findings of 24 epidemiological studies conducted in the last decade. In brief, the incidence rate and risks of LC were several times higher in TB patients in comparison to general or non-TB populations. On the other hand, the incidence rate and risks of TB in LC patients were relatively low. The participants age, sex, smoking status, and medical comorbidities especially respiratory complications were the major confounder that might increase the risk of LC in TB patients, and vice versa. However, observational studies could not guarantee a causal relationship. For example, surveillance bias, classification bias, and detection bias might have been possible because of the frequent hospital visits of the same participants which might have led to a higher detection rate of TB and/or early-stage LC.

Early diagnosis bias and late treatment strategies of LTB might be another responsible factor for high incidence of TB and LC. Most of the studies of current review diagnosed active and inactive TB by chest X-ray (CXR) that may have led to misclassification of participants as having TB since CXR has high sensitivity but poor specificity for the diagnosis of pulmonary TB [56–58]. However, two studies performed TB diagnosis by QuantiFERON-TB Gold In-Tube tests that is known as the gold standard test [47, 52]. Even pulmonary comorbidities may significantly mask symptoms and delay the diagnosis of LC or may even prevent a full diagnostic evaluation with the proper staging of the disease. Before starting the treatment of TB, the possible disease state of LC also should be diagnosed which can prevent the development of LC.

Similarly, a diagnosis of active TB affects the treatment administered to an LC patient. For example, chemotherapy or surgery may be delayed due to a TB diagnosis or associated medication therapy. Previous study reported that the identification of TB infection interrupted the administration of bortezomib in patients with multiple myeloma, which significantly affected patient outcomes [59]. Moreover, LTBI screening at the time of a diagnosis of a high TB risk LC may enable the prevention of active TB during cancer treatment and empower the appropriate administration of chemotherapy. Cancer patients with decreased cellular immunity face an increased risk of active TB and remain in a prolonged state of immunocompromise. Therefore, LTBI screening and treatment are needed to prevent opportunistic infection and should be performed at the time of LC diagnosis simultaneously.

The increased risk of TB development may be confounded by the associations of smoking and excessive alcohol use, which are also independent risk factors for LC [60, 61]. Therefore, it remains controversial whether TB alone can increase the risk of LC. To clarify the question, most of the studies analysed the association between TB and LC after adjustment of smoking status and alcohol consumption. One study which conducted a stratification analysis by smoking status showed that patients with PTB independently had an increased risk of LC [13]. So, it is noteworthy findings and clear evidence of noncasual effect of TB in LC development. However, contradictory result was addressed in one recent study [35]. They reported that never smoker had higher risk of LC compared to the past or current smoker with the history of PTB. However, the study participants were not general populations rather COPD patients. The findings might be due to the higher percentage of never-smoker COPD patients than that of ever smoker. Another possible reason may be the misdiagnosis of COPD in never-smoker participants. Moreover, they addressed the limitations of their findings by mentioning the necessity for confounder adjustment such as occupational or environmental exposures, which are well-known risk factors for COPD and LC development among never smokers [35, 62–64].

Several medical comorbidities may increase the risk of LC in TB patients, and vice versa. One study demonstrated that both male and female TB patients with asthma and COPD had increased risks of lung SqCC, adenocarcinoma, and SmCC [44]. Recent study also showed that COPD patients with history of PTB have higher risk of LC incidence in comparison to without history of PTB, but in non-COPD patients, the difference was null [35]. The results of the study suggest a potential synergism of PTB and COPD in increasing risk for LC, although PTB is the independent risk factors for both COPD and LC [35, 56]. However, the reason why PTB patients without COPD did not develop LC limits their discussion. Furthermore, like COPD, the presence of several baseline chronic inflammatory diseases especially HIV and AIDS may increase the incidence of LC in TB patients, an issue that was not addressed [56]. On the other hand, previous studies stated that patients with other cancer including oesophageal cancer, pancreatic cancer, hematologic malignancy, and head and neck cancer faced a particularly high risk of developing TB [13, 51]. Even histological types of LC, including SmCC and sarcoma, were strongly associated with TB [13]. So, it would be highly appreciating to investigate the incidence and risk of all possible cancer with their histologic types in TB patients in the future. Determining risk factors for specific types of LC can help physicians gain a detailed understanding of the etiology of LC and therefore identify the high-risk population for screening. On the contrary, patients with chronic kidney disease may receive long-term steroid therapy and chemotherapy, which may account for the increased risk of TB [65]. Thus, a study adjusting all possible medical comorbidities is highly expected as few studies did it which strengthens the evidence [12, 41, 55].

The LC incidence rate and risk were found to be higher in men than that of women especially in Asian countries [13, 36, 47]. The reason behind this finding might be explained by the smoking status and tendency. Smoking tendency and frequency in men are higher in compared to women in Asian country; for example, the prevalence of smoking is almost 10-fold higher in Taiwanese men than that in women [66]. Also, male populations were frequently exposed to environmental pollutants than that of their women partners in which some pollutants are strongly associated with respiratory diseases that might regulate TB pathogenesis.

### 4.1. Knowledge Gaps

One of the limitations of the outlined studies is the ethnic background. Majority of the studies were conducted in Asian countries limiting the generalizability of outcomes in worldwide perspectives. Although the number of study participants was significantly high, the overall low number of active TB or LC in comparison to control group might result in limited statistical power of analyses. Some studies recruited self-reported TB patients that causes potential misclassification because not all subjects may remember their respiratory TB, or they may be unaware that they have had TB in the past. TB and LC have been confused and misdiagnosed for years due to similar clinical symptoms of both diseases. Thus, TB might imitate or mask LC. Some studies excluded laboratory results, such as sputum culture, exercise capacity, lifestyle data, nutrition supplements, and family history of systemic disease and specific chemotherapeutic regimens, and the health insurance data utilized did not include the histological stage and severity of primary cancer that may affect the metastatic ability. The possibility of sampling bias in the diagnosis of TB could not be excluded since some studies did not consider comorbidities such as diabetes, malnutrition, asthma, COPD or other lung diseases, HIV infection, renal insufficiency, and immunosuppressant therapies. Some studies did not consider lifestyle-related factors such as obesity, physical inactivity, diet and habits, and family history, which are closely associated with LC [67, 68]. Retrospective studies are at risk of selection bias (cases lost to follow-up) and measurement bias (data obtained from medical records) that may have resulted in a bias in the results too. Few studies had no appropriate control group to compare the incidence and risk of TB or LC and did not find evidence for synergism. Another limitation is unavailable data on the prognostic factor WHO performance status of LC cases. As cancer patients usually receive greater medical attention in terms of frequent examinations, this might contribute to a higher rate of notified TB cases among LC patients. There is a possibility of ascertainment bias through which patients with an index diagnosis are more likely to have another disease diagnosed compared with patients without the index diagnosis.

### 4.2. Future Perspectives

In our summarized studies, some enrolled TB patients are from only one hospital. Therefore, recruitment of baseline patients (TB or LC) national wide or from multiple hospital and clinics will be appreciated. In addition, the selection of control group or general populations should be well defined to avoid the bias. So, in order to evaluate the risk factors of concomitant TB and LC, multicenter and prospective studies are required in the future. Furthermore, the prevalence of TB and LC treatments varies according to the country and the era. Hence, these factors should be considered when adopting or comparing results with those of other countries or previously reported data. Inclusion of the information on the diagnosis of TB and LC, and histologic types of LC, could have strengthened the analyses. The follow-up trajectories of the LC in TB patients or vice versa for several years should have neem conducted to evaluate minimum time required for the transformation of TB to LC or vice versa. Since the risk of LC in TB patients or vice versa depends to a great extent on TB epidemiological situation in certain geographic region, the study should be carefully generalized in countries with low TB or LC incidence. The possible noncausal factors, for example, common genetic or environmental precursor and infectious and noncommunicable diseases which may lead independently to TB and LC, should be considered at baseline and through the follow-up periods. Path analysis might shed light among the relationship of sociodemographic and lifestyle factors, medical comorbidities, environmental chemicals with concomitant TB in LC development, and/or vice versa in the future. Furthermore, investigation of synergistic relationships of TB and potential medical comorbidities especially COPD, asthma or other lung diseases, HIV and other microbes in the development of LC is highly recommended in large-scale cohort. The mechanistic overview of how TB patients develop LC in patients with these comorbidities should also be addressed in the future. Finally, follow-up screening of TB and LC should continuously assess and ensure the modern diagnosis and treatment strategies to prevent secondary form of diseases and to manage long-term respiratory complications due to TB survival [69–71].

## 5. Conclusion

Our summarized studies explicitly suggest that existing TB can increase the incidence rate and risk of LC, and vice versa. The patients' age, sex and medical comorbidities might influence the relationships. Large prospective cohort study is warranted exploring the deep scenario in the future.

## Figures and Tables

**Table 1:** The summary of incidence and risk of LC at TB patients, and vice versa.

Ref.	Study design (country)	Study subjects, age (*n*)	Follow-up (*y*)	Outcome	Diagnosis and/or identification	Covariate	Key findings
[35]	Cohort (South Korea)	Adults; 50-84 y (COPD = 13165 and non-COPD = 34467)	13	LC	TB: CXR LC: ICD-10, C33, or C34 code	b, c, d, m, and ag	(i) Observed 430 incident cases of LC (IR = 524/100000 person/year) in participants without PTB and 148 cases in those with PTB (IR = 931/100000 person/year) after a median follow-up of 7.7 y (370617 person/year) in COPD group (ii) The risk of LC was significantly higher in COPD patients with PTB in comparison to COPD patients without PTB (sub − aHR = 1.24 (1.03, 1.50)) (iii) The non-COPD group had 437 and 98 incident LC cases in participants without and with PTB, respectively (IR = 188 vs. 241/100000 person/year). The incidence risk of LC in non-COPD patients with and without PTB was not different (sub − aHR = 0.98 (0.78, 1.22)) (iv) The association of PTB history and LC development was more evident in never smokers with COPD (aHR = 1.42 (1.04, 1.95)). In contrast, among participants without COPD, the incidence risk was null (aHR = 1.13 (0.89, 1.44)) (v) Found null associations among PTB, smoking status, and COPD (*p* = 0.17)

[49]	Cross sectional (Egypt)	Adults; mean age: 63 (64)		LTB	LTB: QuantiFERON-TB Gold In-Tube tests LC: bronchoscopy, CT, and US-guided biopsy	N/A	(i) Latent TB was detected in 16 (25%) patients, while 48 (75%) had negative results of the QFT-GIT test (ii) Age and sex were not associated with LTB (*p* > 0.6 and 0.14, respectively), but a current smoker was associated with a higher prevalence of LTB (*p* < 0.001) (iii) Medical comorbidities, tumor site, and histopathology were not associated with latent TB

[12]	Cohort (Taiwan)	Adults; ≥20 y (TB = 693, without TB = 13868)	15	LC (2nd)	TB: CXR LC: ICD-10, C33, and C34 code	a, d, g, h, and i	(i) The risk of secondary LC was 1.671 times greater in the TB cohort than in the non-TB cohort (aHR = 1.67 (1.525, 1.832)) (ii) Compared with the local hospital, the risk of secondary LC was higher in the medical center (aHR = 2.33 (1.93, 2.82)) and in the regional hospital (aHR = 1.73 (1.44, 2.07)) (iii) The 1-, 5-, 11-, and 15-year actuarial rates of secondary LC were 6.89%, 10.42%, 10.96%, and 10.97% in the TB cohort and 4.27%, 8.18%, 9.05%, and 9.10% in the non-TB cohort, respectively

[50]	Cohort (Canada)	Migrant adults; ≥15 y (10006)	19	Active TB	TB: tuberculin skin test and IF-gamma release assay LC: CXR, CT, and PET biopsy	a, b, j, k, and ad	(i) Study participants with LC had the highest risk of active TB (aHR = 11.20 (7.40, 16.90))

[36]	Cohort (South Korea)	Adults; ≥20 y (PTB = 3,776; control = 18, 880)	11	LC	PTB: sputum microscopy with or without culture LC: CT scan	a, b, c, and d	(i) The IRR in the pulmonary TB group was 12.26 within 1 year and 3.33 at 1–3.9 years after TB infection, compared to the control group (ii) There was an increased risk for LC in pulmonary TB patients compared to controls (aHR = 4.18 (3.15, 5.56)) (iii) Compared to patients < 50 years of age, the risks for LC were HR 9.85, 7.1, 3.32, and 2.57 in patients aged 50–59, 60–69, and ≥70 years, respectively (iv) The risk for LC was higher in men than in women (aHR = 2.35 (1.60, 3.46)) and in current smokers than in never smokers (aHR = 2.00 (1.43, 2.78))

[51]	Retrospective cohort (18y) (South Korea)	Adults; ≥20 y (LC = 34783; C1 = 69566; C2 = 1151402)	15	All TB (CDTB and BCTB)	TB: ICD-10 codes A15–A19 LC: CT scan, PET scan	c, e	(i) The BCTB IR was 577 and 37/100,000 person-years in cancer patients and controls, respectively (ii) The IRR of BCTB was 14.30 (all cancer cases) and 50.35 (iii) The LC IRR for the development of all TB and BCTB compared to the control cohort 1 (C1) was IRR = 42.85 (37.06, 49.54) and IRR = 50.35 (42.22, 60.06)], respectively, and to control cohort 2 (C2) was IRR = 38.95 (34.84, 43.54)

[52]	Retrospective cohort (Thailand)	Adults; median age: 60 y (40948)	18	TB	TB: ICD–10 version 2016, code A15.0–A19.9 LC: ICD–O 3^rd^ edition, code C00.0–C80.9	a, b, and l	(i) Cumulative incidence = 1.15%, IR = 421.86 cases per 100,000 cancer patients per year (ii) Compared to thyroid cancer, TB infection was more associated with LC without histopathological confirmation (aIRR = 6.22 (2.57–15.04))

[13]	Cohort (South Korea)	Adults; ≥40 y (20252)	6	LC	TB: CXR LC: ICD-10 diagnosis code C33 and C34.	a, b, c, d, f, m, and n	(i) The overall relative risk of LC for patients with old PTB was RR = 5.66 (3.17, 10.12) compared to the control group (ii) The RRs of LC in men and women with old PTB were 5.28 (2.65,10.50) and 4.06 (1.19, 13.89) compared to the control group (iii) For under 60 years aged patient, RR was 2.19 (0.53, 9.12) and for over 60 years aged patient 5.51 (3.03, 10.01) (iv) RR of LC for never smokers, ex-smokers, and current smokers with old PTB was 4.82 (1.60, 14.56), 3.56 (1.13, 11.16) and 7.10 (2.94, 17.13) (v) The RR of people with old PTB for squamous cell carcinoma, adenocarcinoma, and other types of LC was 3.39 (0.95, 12.05), 4.32 (1.77, 10.58), and 13.07 (4.96, 34.50) (vi) The aHR was 3.24 (1.87, 5.62) compared to controls. For never smokers and current smokers, the aHRs of LC were 3.52 (1.17, 10.63) and 3.71 (1.49, 9.22) and for ex-smokers was 2.16 (0.89, 5.24) compared to controls

[37]	Cohort (Lithuania)	Adults to elders; 25–75 y (21986)	Mean 6.3	LC	TB: bacteriological or histological confirmation via microscopy LC: CT scan, PET-CT scan	a, b, c, e, f, o, and p	(i) Compared with the general population, the SIR of LC among TB patients was 3.83 (3.49, 4.19). We observed statistically significantly increased risks in both smokers (SIR = 4.48 (4.04, 4.96)) and nonsmokers (SIR = 1.93 (1.56, 2.36)) (ii) The risk of LC increased with age (aHR = 6.36 (3.53,11.44) and aHR = 14.22 (7.89, 25.66) for 45-55 and ≥55 y compared to ≤45 y, respectively) and was higher in smokers (aHR = 4.42 (2.47, 7.89)) then nonsmoker, and in respiratory TB (aHR = 1.88 (0.86, 4.11)) and then nonrespiratory TB

[47]	Cohort study (South Korea)	Adults; ≥20 y (1607710)	16	LC	TB: CXR LC: chest computed tomography	a, b, c, and q	(i) The presence of underlying TB was significantly associated with increased risk for LC incidence (HR = 1.37 (1.29, 1.45)) in men and (HR = 1.49 (1.28, 1.74)) in women and mortality (HR = 1.43 (1.34, 1.52)) in men and (HR = 1.53 (1.28, 1.83)) in women in comparison with without TB participants (ii) Similarly, ex-smoker and current smoker had higher incidence and mortality risk for increased LC in comparison with never smoker

[42]	Cohort (Taiwan)	Adults; ≥20 y (15219024)	5	LC	TB: ICD-9-CM codes 010–012 and 137.0 LC: ICD-9-CM code 162 or ICD 10 codes C34.0, C34.1, C34.2, C34.3, C34.8, and C34.9	a, b, c, d, g, and r	(i) In men and women, the aHR of SqCC were 1.37 (1.21, 1.54) and 2.10 (1.36, 3.23), respectively, for TB (ii) The aHR of adenocarcinoma and small-cell carcinoma was 1.33 (1.19, 1.50) and 1.86 (1.57–2.19) and 1.24 (1.01, 1.52) and 2.23 (1.17, 4.25), respectively, for TB (iii) A significantly high incidence of LC in male patients with TB (HR = 1.35 (1.26, 1.44)) was observed (iv) The risk of LC was high in female patients with TB (HR = 1.97 (1.73–2.24)) (v) Found increased risk for histological types of LC depending on increased age of participants with TB

[44]	Cohort (Taiwan)	Adults; ≥20 y (5406)	5	TB	TB: ICD-9-CM: 010–012, 137.0 SqCC: ICD-9-CM 162 or ICD 10, C34.0, C34.1, C34.2, C34.3, C34.8, and C34.9	N/A	(i) For all cause-mortality, HR was 1.14 (1.00, 1.31) for individuals with TB (ii) TB increased risk for mortality in patients with SqCC with rate ratio of 1.21 (1.20, 1.22)

[53]	Cohort (Japan)	Adults; mean age 69.9 y, 963	6	TB	TB: CXR LC: CT scan	N/A	(i) The cumulative incidence of TB among LC patients at 0.5, 1, and 2 years was 0.65%, 1.15%, and 1.38%, respectively

[39]	Cohort (Denmark)	Adults; median age 43.4 y (15024)	18	LC	TB: microscopy LC: CT scan, bronchoscopy	a, b, c, and e	(i) All-time (median follow-up 8.5 years) SIR for LC was 3.40 (3.09, 3.74) (ii) The 3-month SIR for LC was 40.9 (34.0, 49.0), and 0-1-year SIR was 16.87 (14.50, 19.51) (iii) A 2.24-fold (1.88, 2.64) increased risk beyond 5 years for LC following respiratory TB

[54]	Cross sectional (Taiwan)	Adults; ≥ 20 y (LC = 340; non − LC = 391)	1	LTB, non-LTB, and intermediate TB	TB: QuantiFERON-TB Gold In-Tube (QFT-GIT) LC: chest computed tomography	a, b, and c	(i) Independent factors associated with LTB in LC patients included COPD (OR = 2.41(1.25, 4.64)), main tumor located in typical TB areas (OR = 2.02 (1.15, 3.55)), and fibro calcified lesions on chest radiogram (OR = 2.73 (1.45, 5.11)) (ii) The proportion of concomitant LTB when LC diagnosis was significantly higher in patients who developed active TB than those without during the follow-up period (OR = 9.26 (1.02, 83.99))

[48]	Cohort (South Korea)	Adults; 40-59 y (7009)	Mean 8.5	LC	TB: CXR LC: ICD-10 diagnosis code C33 and C34	a, s	(i) The aRR of PTB history of current smokers in LC was 1.85 (1.08, 3.19) (ii) The aRRs of past medical history of PTB in occurring LC for smoker who smoke for >31 years (aRR = 2.00 (1.06, 3.79)) and who smoke > 21 cigarette per day (aRR = 2.05 (1.09, 3.83))

[46]	Retrospective cohort (China)	Adults; mean age 60 y (782)	5	LC	TB: chest CT scan LC: bronchoscopy	a, b, c, t, and u	(i) The median survival of SqCC patients with TB was significantly shorter than that of patients without TB (1.7 vs. 3.4 years, *p* < 0.01) (ii) The presence of an old PTB lesion is an independent predictor of poor survival with an HR of 1.72 (1.12, 2.64) in the subgroup of SqCC patients studied

[45]	Retrospective cohort (China)	Elder; mean age 72 y (64574)	11	LC	TB: CXR LC: CXR	a, b, e, f, m, v, w, x, y, z, ac, and ae	(i) TB was consistently associated with a two- to threefold risk of LC mortality (ii) TB remained an independent predictor of LC death (aHR = 2.01 (1.40, 2.90))

[38]	Cohort (Taiwan)	Adults; >20 y (6699)	10	LC	TB: Acid-fast smear and positive culture for TB, CXR (ICD-9-CM code 010.x to 018. x.) LC: CXR, CT scan	N/A	(i) The SIRs for LC among patients with TB diagnosis were 4.09 (3.48, 4.78) for total, 4.09 (3.42, 4.84) for male, and 4.13 (2.67, 6.10) for female (ii) The SIRs for LC stratified by time after TB diagnosis were 12.39 (9.90, 15.33) at <1 y, 2.21 (1.57, 3.02) at 1-5 y, and 1.21 (0.58, 2.23) at >5 y for male. Similarly, 17.24 (10.67, 26.36) at <1 y, 0.95 (0.20, 2.77) at 1-5 y, and 0.60 (0.02, 3.34) at >5 y for female

[43]	Cohort (Netherlands)	Adults; ≥55 y (7983)	18	LC	TB: ICD-10 diagnosis code A15–A19 LC: ICD-10 diagnosis code C33 and C34	a, b, c, f, l, aa, and af	(i) The crude HR of history of TB shows a 1.75-fold increased risk (1.0, 3.1), and the aHR of history of TB shows a 2.36-fold increased risk (1.1, 4.9) with a mean difference of 311 days (ii) The cumulative survival rate of LC patients without TB was higher in comparison to LC patients with TB throughout the follow-up period

[28]	Cohort (Finland)	Adult male smoker; median age 57 y (29133)	20	LC	TB: CXR LC: CXR	a, c	(i) TB was associated with a 2-fold elevation in risk of LC (HR = 1.97 (1.46, 2.65)) (ii) The LC risk was greatest in the two-year window after TB diagnosis (HR = 5.01 (2.96–8.48)) but continued to be elevated at longer latencies, with a 50% increased risk of LC in the overall period two or more years after TB diagnosis (HR = 1.53 (1.07–2.20)) (iii) Both incident and prevalent TB were found to be associated with LC risk (HR = 2.05 (1.42, 2.96) and HR = 1.82 (1.09, 3.02), respectively) (iv) The risk of LC 0–1.9 years, 2–9.9 years, and 2+ and 10+ years after TB diagnosis was aHR = 5.0, aHR = 1.4, aHR = 1.53, and aHR = 1.7

[55]	Retrospective cohort (Taiwan)	Adults; mean age 60 y (cancer = 16487, control = 65948)	8	TB	TB: CXR LC: ICD-9-CM code (162) or A code (A101)	a, b, and h	(i) A crude IRR for active TB 1.68 (1.42, 1.98) and the aHR was 1.67 (1.42, 1.96) (ii) The average interval from diagnosis of LC to diagnosis of active TB was 748 days (644, 852)

[40]	Cohort (Taiwan)	Adults; median age 58 y (TB = 5657, control = 23984)	10	LC	TB: ICD-9-CM (code (010-012, 018) or A code (A020, A021) LC: ICD-9-CM code (162) or A code (A101)	a, b	(i) The IRR of LC was significantly higher in the PTB patients than that in controls (IRR = 1.76 (1.33-2.32)) (ii) Compared with the controls, the IRRs of LC in the TB cohort were 1.98 at 2 to 4 years, 1.42 at 5 to 7 years, and 1.59 at 8 to 12 years after TB infections (iii) PTB infections (HR = 1.64 (1.24-2.15)) and COPD (HR = 1.09 (1.03-1.14)) to be independent risk factors for LC

[41]	Cohort (Taiwan)	Adults; ≥20 y (TB = 4480, Control = 712392)	9	LC	TB: ICD-9-CM of 011 and A-code of A020 LC: ICD-9-CM of 162 and A-code of A101	a, b, h, and ab	(i) The incidence of LC was approximately 11-fold higher in the cohort of patients with TB than non-TB subjects (26.3 versus 2.41 per 10,000 person-years) (ii) An HR of 4.37 (3.56, 5.36) for the TB cohort after adjustment for the sociodemographic variables or 3.32 (2.70, 4.09) after further adjustment for COPD, smoking-related cancers (other than LC), etc. (iii) The HR increased to 6.22 (4.87, 7.94) with the combined effect with COPD or to 15.5 (2.17, 110) with the combined effect with other smoking-related cancers

Ref.: reference; n: number of study participants; y: follow-up time in year; TB: tuberculosis; LC: lung cancer; QFT-GIT: QuantiFERON-TB Gold In-Tube; CXR: chest X-ray; aIRR: adjusted incidence rate ratio; aSIR: adjusted standardized incidence rate; aHR: adjusted hazard ratio; aRR: adjusted relative risk; aOR: adjusted odd ratio; BCTB: bacteriologically confirmed TB; CDTB: clinically diagnosed TB; PTB: pulmonary TB; LTB: latent TB; SqCC: squamous cell carcinoma; N/A: not available. ^a^Age; ^b^sex/gender; ^c^smoking/; ^d^monthly income/household income; ^e^alcohol consumption; ^f^education; ^g^urbanization level; ^h^medical comorbidities; ^i^hospital level; ^j^time since arrival in BC; ^k^high-risk medical comorbidities and TB incidence in country of origin; ^l^cancer type/histological subtype; ^m^BMI; ^n^physical activity; ^o^employment; ^p^family status; ^q^preexisting TB; ^r^geographical area; ^s^intake of coffee and tomatoes; ^t^tumor stage; ^u^surgical approach; ^v^language spoken; ^w^housing situation; ^x^public means-tested financial assistance status; ^y^chronic obstructive pulmonary disease and/or asthma; ^z^family history of malignancy among never smokers and ever smokers; ^aa^smoking pack per year; ^ab^occupation; ^ac^passive smoking; ^ad^immigration classification; ^ae^marital status; ^af^disease stage; ^ag^the Charlson comorbidity index. Note: all values of HR, RR, IRR, SIR, and OR were presented as 95% confidential interval (lower and upper).

## Data Availability

The data that support the findings of this study are available from the corresponding author upon reasonable request.
